# Control, power, and responsibility: a qualitative study of local perspectives on problem drinking in Peruvian Andean highlands

**DOI:** 10.1186/s12992-021-00758-5

**Published:** 2021-09-19

**Authors:** Sakiko Yamaguchi, Raphael Lencucha, Thomas G. Brown

**Affiliations:** 1 Department of Psychiatry, Division of Social and Cultural Psychiatry, McGill University, Montreal, Canada; 2 School of Physical and Occupational Therapy, McGill University, Montreal, Canada; 3 Department of Psychiatry, McGill University, Montreal, Canada; 4 Department of Community Health Sciences, University of Sherbrooke, Quebec, Canada

**Keywords:** Alcohol use, Peruvian Andes, Control, Responsibility, Problematization, Neoliberalism, Ethnography, Market

## Abstract

**Background:**

Alcohol control has emerged as an important global health challenge due to the expanding influence of alcohol companies and limited control measures imposed by governments. In the Peruvian Andean highland, the ritual function of collective drinking is reported to have been weakened in response to the increased availability of alcohol and the experience of political violence. This study seeks to merge the broader political economy with local experience and culture to provide a deeper understanding of the dynamic between global processes and local realities.

**Methods:**

We used purposive sampling to recruit participants. We conducted in-depth interviews (*n* = 28) and focus group discussions (*n* = 19) with community participants, teachers, health workers, alcohol vendors and police officers. Thematic analysis identified patterns of individual and collective meaning situated in relation to social, political and economic factors.

**Results:**

Local perspectives and behaviour regarding loss of control over alcohol are shaped through the complex patterns of power and meaning exerted and experienced by different actors. Participants’ emphasis on parents’ lack of control over alcohol use by “abandoned” children reflects the structural vulnerability of some Andean families struggling with economic hardships. Participants also emphasized how alcohol consumption was tied to forms of control exerted by men in households. Participants expressed that some men demonstrated their masculine identity and symbolic power as the breadwinner through spending on alcohol. The third emphasis was tied to the market economy. Participants expressed that the expansion of the alcohol market and perceived absence of government control coupled with macroeconomic conditions, like poverty, shaped patterns of alcohol consumption.

**Conclusion:**

Our findings illustrate how problem drinking is shaped not simply by an individual drinker’s lack of self-control but also by a regulatory environment that enables the unrestrained marketing of alcohol products and the creation of a culture of consumption. Harmful consumption is mediated by the reshaping of the Andean cultural practice of collective drinking. Attending to local perspectives is essential for policies and interventions that connect structural dynamics with the cultural and experiential aspects of alcohol consumption.

## Introduction

The 2018 Global Status Report on Alcohol and Health indicates that alcohol use results in approximately 3 million deaths per year and numerous other social problems [1]. Some global health scholars suggest that alcohol remains a ‘blind spot’, not receiving adequate attention in research and prevention efforts given the high burden on global mortality and morbidity [2, 3]. Calls for increased attention to alcohol as a globalized health issue are supported by a growing body of research demonstrating the aggressive approaches taken by transnational alcohol firms to expand their global market while at the same time lobbying governments to ensure minimal or no regulation [4, 5]. The globalization of industry influence is propelled by the increasing consolidation of market control among large companies. For example, Jergin and Ross found that the share of the beer market held by the top 10 companies increased from one quarter in 1980 to two thirds by 2017 [6]. This context leads to concern over the growing impact of the alcohol industry, as a commercial determinant of health, in low- and middle-income countries (LMICs) [7]. While some regions like Europe have seen a decline in alcohol use, others like Africa and Asia have seen an increase in consumption [1].

Amplifying the risk of alcohol company influence is the fact that over half of the governments in the world do not have one health protective policy in place to control the alcohol market [1]. Weak or non-existent alcohol control is sustained through industry pressure in many countries [2, 7, 8]. Local governments are also attracted by alcohol revenues, encouraging a tolerance for high levels of alcohol-related harm [9]. Transnational alcohol companies actively promote foreign alcohol products and increased consumption with a message of modernity and prosperity [9]. In many LMICs, these developments have resulted in measurable increases in the volume of alcohol consumption per occasion, and the frequency and reasons for drinking [9]. As foreign alcohol products represent a new social status symbol, they also attract economically vulnerable populations. As noted, evidence suggests that promotion of transnational alcohol beverages in LMICs can alter social and cultural norms that can play a role in moderating or restricting alcohol consumption [10]. This shift away from traditional norms of alcohol use that is culturally rooted and often restrained can produce new ways of framing the ideas of responsibility and control.

While the emphasis on universal harms, industry practices, and best practices for alcohol control is critically important to alcohol control, there is also a need to understand how these universals intersect with local understandings and practices. This understanding can lead to a more sensitive and responsive approach to alcohol control that respects the unique ways in which global processes influence cultures of consumption, and how these cultures insulate from or respond to global processes.

We know that the problem of harmful alcohol use has been conceptualized using different points of emphasis and epistemic frameworks specific to the time and culture, pointing to the need for a localized understanding of global processes [11, 12]. For instance, during the late 18th to mid-nineteenth century in the US, where the temperance movement emerged, alcohol became problematized as a transgression of personal self-control, independence, and productivity [11, 13, 14]. In the current era where neoliberal rationality both dominates and creates contradictions in the relationship between market, state and society [15], the aim of moderate alcohol consumption and self-control over drinking fostered by public health actors sits in tension with the perceived economic benefits of excessive consumption for the industry and state. Both examples illustrate that responsibility and control are often located with the individual drinker, with implications for how alcohol is controlled [12, 16].

Neoliberal rationality is a mode of governing that aspires to produce self-regulating citizens [17]. This mode of governing also releases government from making value judgements about the personal and community consequences of alcohol supply on consumers; alcohol is viewed as a neutral economic commodity like others [18]. In this context, the neoliberal discourse stresses individual health management by self-control rather than market regulation to promote healthy choices, thus producing moral narratives of personal responsibility and blame [12, 16, 19–22]. Individuals showing drinking patterns that are considered harmful to their health and society at large are characterized as irrational and weak. Consequently, the trope of “lack of control” gives a means of expressing power and rationality when drinking is controlled [23].

The need to address harmful alcohol use is relevant to Ayacucho, a region located in the south-central Andean highlands of Peru. While *chicha* (fermented corn beer) has been traditionally consumed at a ritualistic part of community fiestas since the pre-Spanish conquest period, beverages with high alcohol concentration such as *aguardiente de caña* (sugarcane spirit) became available with the introduction of distillation in the sixteenth century, followed by commercialization of beer in the nineteenth century (Pedersen D: Estudio sobre patrones de consumo de alcohol en poblaciones urbanas y rurales de la sierra (Ayacucho, Peru), unpublished). Ethnographic studies in Andean highlands have documented collective intoxication as a cultural phenomenon that is associated with Quechua identity, Andean history and socio-cultural context [24, 25]. The culturally embedded drinking patterns appeared to shift during the 20-year political violence (1980–2000) that hit the Ayacucho region the hardest. Medical anthropologist Kimberly Theidon, who conducted ethnographic research in various communities throughout rural Ayacucho between 1995 and 1999, opined that the rural *campesinos* (peasants) had “learned to drink” during this period [26]. The ritualized form of drinking during fiestas appeared to transform into first a means of coping with the constant fear of death, and then alcohol dependence after the conflict. Ayacucho experiences the highest prevalence of alcohol use disorder (AUD) in Peru [27]. Today, the WHO/PAHO working group recognizes the alcohol industry as a key determinant of alcohol consumption and related harms in this region and globally [7]. This group has called for policies to control alcohol consumption through a range of health information and promotion programs [28]. Currently, there is no written national policy on alcohol control, which underlies the absence of legally binding regulations on alcohol advertising and sales promotion in Peru [1].

This ethnographic study integrates political economy analysis into cultural and medical anthological approaches to understand individual and community alcohol-related practices in the south-central Andean highlands of Peru. The present analysis is guided by the following questions: “What are the local perspectives on problem drinking?” and “How is the problematization of alcohol use in the community shaped by Andean cultural practices, meanings, and values associated with drinking?” In this paper, problem drinking is used to cover a range of alcohol use patterns that increase the risk of physical and/or mental health harms (including alcohol use disorder) and have negative impacts on the safety and well-being of others. By exploring local understandings of problem drinking, this paper aims to highlight: 1) interactions and tensions between socio-economic structures and social processes and individual and collective meaning; and 2) how culture intersects with the power of different actors involved in production, distribution, and consumption of alcohol.

## Methods

### Setting

This study was conducted in two districts (Carmen Alto and Socos) in the region of Ayacucho located in the south-central part of Peru. Carmen Alto is a semi-urban district adjacent to the City of Ayacucho, the capital city of the region, with a population of 21,350 residents. Seventy-four percent of the population are Catholic whose saints are celebrated through occasions of collective alcohol use lasting 3 to 4 days. The remaining 26% are Evangelical, a doctrine that prohibits alcohol use [29]. The major occupations involve work in small and medium-sized businesses such as restaurants, small convenience stores, internet cafes, bakeries in the urban area, while residents in rural areas of the district engage in animal husbandry and subsistence farming [29].

In relation to alcohol use, Carmen Alto has a long history of *arriero* (muleteers) who used mules to commercially transport and exchange agricultural products such as coca, coffee, cacao and the daily necessities in wide areas covering *Selva* (Amazonian jungle) and *Costa* (Coastal) areas from late sixteenth century [29]. In addition to the Catholic festivals, a common ritual at the time involved the consumption of *caña* (sugarcane alcohol) by friends, godchildren, and godparents to bid farewell to the *arrieros* who were departing to faraway places. With the improvement of transportation and the road infrastructure in the 1970s, *arriero* was no longer needed.

When the violence intensified during the period of political conflict, many villagers and their families in the rural areas of the Department of Ayacucho were displaced to Carmen Alto. When the political violence ended, *pandillas* (youth gangs) began to be seen engaging in heavy alcohol use, usually *caña* or other liquor mixed with soda, or *chicha* (fermented corn beer). This was attributed to disruptions in relations of rural migrant families who had fled intensified violence [30].

As internal migration in the pursuit of better economic opportunities continued, migrants largely residing in less developed rural areas with limited access to water, sanitation, and electricity now account for 59% of district residents [29]. Carmen Alto has one entertainment zone close to the district centre. In contrast to quiet weekdays, *discotecas* and *recreos*, popular drinking places are very busy and packed on weekend nights. They serve food and alcoholic (and non-alcoholic) beverages mainly beer day and night, while bands perform live music on an open stage. On the streets, some female vendors also sell hot drinks made with *caña* or alcohol mixed with herbal infusions to give warmth during the cold nights in the Andean highlands.

Socos is a semi-rural district 18 km away from the capital city of Ayacucho, where the majority of the population of 7108 engage in subsistence farming and animal husbandry [31]. Unlike Carmen Alto, there are no bars or discos, however alcohol beverages, largely beer and *caña*, can be purchased at small convenience stores and are served at casual diners. While beer is consumed at community fiestas and social gatherings, similar to Carmen Alto, *caña,* which is cheap and known to be harmful to health, is often consumed by local farmers who bring a bottle to *chacra* in the morning.

### Ethical oversight

The Douglas Mental Health Institute Research Ethics Board (IUSMD-15-43) and the Cayetano Heredia Peruvian University Institutional Ethics Committee (66752) provided ethics approval and oversight of the study protocol. In addition to procedural ethics, trust building with study participants was challenging in a context where during times of violence disclosing identities could be a fatal act and mistrust became a survival strategy. Facing an omnipresent fear over the uncertainty of the use of personal accounts and reluctance to disclose personal information, the local research consultant played a significant role in clarifying the use of generated knowledge and navigating power asymmetry between the foreign researcher and local participants using the shared Quechua language and identity. The local research consultant and the first author consulted actively about how positionalities characterized by the particular attributes of the researcher and of the research consultant such as nationality, language, and gender may have influenced the interactions with the study participants [32].

### Participant recruitment and final sample

The first author, who had previously worked on a project for improvement of mental health services for victims of political violence, collected qualitative data using key informant interviews, focus group discussions and participant observation (July–November 2016). The observation further took place in the subsequent years (June–September 2017, March–April 2019). A local research consultant, Mr. Julián Berrocal Flores (JBF), who is bilingual in Spanish and Quechua and has experience as a research assistant for various foreign researchers, not only functioned as an interpreter but also as a cultural broker who negotiated the access to study participants and facilitated the process of knowledge co-construction.

The participant inclusion criteria were: 1) a minimum age 18 years, and 2) verbal communication abilities in either Quechua or in Spanish. We approached local police stations, secondary schools, and health centers of the study sites to recruit interview participants. Our inquiries at the municipality level identified potential interview participants who were knowledgeable in the history and socio-economic characteristics and traditions of the district. Table 1 categorizes the final 28 interview participants (Socos *n* = 12, Carmen Alto *n* = 16). JBF and the first author carried out interviews with key informants.

**Table 1:** Number, role and provenance of interview participants

	Socos		Carmen Alto	
	Male	Female	Male	Female
Community informants	2	3	2	2
Local police officers/security officers*	0	0	2	2
Secondary school teachers	1	1	1	1
Health professionals	1	1	1	2
Alcohol vendors/*chicha* (traditional fermented corn beer) producers	1	2	1	2
Total	5	7	7	9

^*^Socos had no police services (i.e., no recruitment of police officers/security officers)

In order to recruit focus group participants, we contacted secondary schools, health centers, and the leaders of established community-based organizations (Mothers’ Club, neighbourhood council, sports teams) in Carmen Alto and a group of community health promoters in Socos. Table 2 presents characteristics of the 19 focus group discussions. On average, eight participants per group took part in discussions (Total 148 participants: Carmen Alto female = 43, male =24, Socos female = 42, male = 39). We used interview guides that probed participants’ views on common drinking practices and related cultural and social values, and current health and social issues seen in each district. We posed questions such as “What does the term *borrachera* (intoxication) bring to your mind?”, “Based on what you have heard or seen, what motivates people to drink alcohol?” and “What does alcohol bring to your community?”. Key informant interviews and focus groups lasted between 60 and 90 min; one focus group with male participants in Carmen Alto lasted 30 min due to the weather conditions.

**Table 2:** Number of focus groups by participant type and location

Group	Socos	Carmen Alto
Secondary school teachers	1	1
Health professionals	2	1
Community participants (male)	3	2
Community participants (female)	3	4
Community participants (mixed)	0	2
Total	9	10

Prior to each focus group and interview, JBF first asked candidates what language they preferred, and verbally explained the study purpose and the activity content, confidentiality issues including data management, participants’ freedom to withdraw at any time, and compensation. Once candidates agreed to participate and understood that interviews and focus groups would be audio-recorded, they were asked to provide their consent either in written or verbal form. Participants received compensation consisting of a package of food or stationery at the end of the activity.

The first author conducted participant observation, documenting daily communications with local people, and observations of daily activities and local customs, cultural/religious events, and drinking venues such as *recreos* on weekends throughout the eight-month fieldwork. The first author also participated in cultural practices by drinking, eating, and dancing with people at *fiesta patronal* and *fiesta de agua* in Socos, neighbors, and JBF’s family members during birthdays and community festivals. These interactions contributed to building rapport with community members in a cultural context in which alcohol use fosters relatedness. It also helped understanding of drinking behaviours during specific occasions, such as when some females drink to intoxication during the community fiestas, that would not have been possible otherwise.

### Translation of materials

The self-identification of the Ayacucho population is 81.2% Quechua [33]. The back-translation method established the cultural and linguistic validity of research materials. The bilingual research consultant (Spanish-Quechua), JBF, first translated the Spanish versions of all interview guides to Quechua. Another bilingual translator conducted back translation to Spanish, then JBF, the translator, and four local bilingual volunteers checked semantic, technical, and content equivalence between versions [34]. Discrepancies between the original Spanish and the back-translated Spanish items were discussed until agreement on the equivalent Quechua version was reached. In Carmen Alto, participants chose to conduct interviews in Spanish or mixed Spanish and Quechua, as is typical in their daily lives. In Socos, Quechua was the primary choice of participants, except for the health professionals and high school teachers who chose Spanish.

### Analysis

The recordings were transcribed and analyzed in Spanish. For data collected in Quechua (*n* = 14), transcribed data were translated into Spanish. Thematic analysis was used to categorize initial inductive themes such as “motives of alcohol use,” “role of advertisement,” “male drinking,” “alcohol availability.” As *“No hay control”* (“There is no control”) was the recurrent expression used to describe drinking patterns in the community, different aspects of lack of control were explored to identify patterns of meaning associated with ‘control’. The thematic analysis was guided by the approach of Braun and Clarke [35], which involves an iterative process of reading transcripts, deductive and inductive coding, categorizing of codes, engaging with the literature, and further reviewing and refining coding scheme [36, 37].

During the analysis, the political economy approach proposed by medical anthropologist Merrill Singer helped to explore the multi-level influence of historic and political-economic forces on the social determinants associated with problem drinking [38, 39]. This approach was used to shed light on how institutional and structural factors, such as capitalism, economic inequalities, corporate activities, class hierarchy, race, and gender shape alcohol use practices and their implications for personal suffering, negative health outcomes, and society as a whole [38–40]. More specifically, the concept of structural vulnerability scrutinizes how social forces beyond individual control constrain personal behaviours, in contrast to strictly individualist conceptions that attribute willful and moral irresponsibility to ‘problem drinkers’ [41]. While a political economy approach provides an important orientation towards structure, an exclusive focus on structure may mask individual and collective meanings of suffering and intersubjective experiences [42]. This is why individual subjectivity, social and cultural practices, and individual and collective agency were considered in relation to structural conditions in this study [43].

Qualitative data analysis software Atlas ti®8.4.24 was used to manage and organize the data. During the analysis, the first author consulted with JBF to examine the cultural salience of alcohol-related values highlighted in the interviews and to make sense of observed behaviours within the historical and socio-cultural context of the south-central Peruvian Andean highlands. Interview and focus group data were triangulated with fieldnotes and observations. In addition, emerging themes were discussed with co-authors TB and RL to further consider how macro-political and economic structures are linked to localized understanding of alcohol use. Some preliminary findings were shared with community participants in 2017 and 2019 to validate my interpretations of their accounts. While they generally agreed with the presented interpretation, they also stressed their view that the environment surrounding alcohol use was rapidly changing and that alcohol-related problems need to be addressed at a community level.

## Results

As a psychologist in Carmen Alto explained, culturally embedded collective alcohol use through community fiestas and social gatherings with family, friends, and co-workers on the weekend has normalized *borrachera* (intoxication). Participants presented the perceived benefit of controlled alcohol use, such as enhancing social interaction by putting away *vergüenza* (embarrassment), *temor* (fear), and *miedo* (fear) and facilitating talk, song, and dance. In one *recreo* in Carmen Alto, groups of men and women, who were initially sitting, chatting cheerfully and drinking, started to leave their tables and start dancing while holding a glass or a bottle, singing loudly with the songs played live by music bands. Traditional *huayno* (Andean folklore music) in combination with the effect of alcohol bring high spirits, light rhythms and fast tempos to dancing during community fiestas and social gatherings at home, bars, and *recreos*. Some *huayno* describe the beauty of traditions and cultural identity [44]. In the cold harsh Andean environment, alcohol also gives the feeling of warmth and energy and is seen to motivate day laborers to accomplish physically demanding work in the *chacra* (field). Likewise, alcohol is seen as an enhancer of achieving a certain objective in negotiations. Offering alcohol in discussions was explained as symbolic presentation of sincerity, trust, and commitment to build close relationships, and believed to facilitate the negotiation of important issues in business and marriage.

Another pattern of problem drinking is using alcohol to cope with daily struggles such as family problems and financial problems, and emotional suffering from relationship problems, which may lead to a bad habit (*vicio*). Drinkers who consume alcohol to cope are largely seen as lacking control over the amount of alcohol consumed as well as emotional outbursts. Many participants noted that this type of drinking often occurred alone, which is considered as a transgression of cultural practice of sharing alcohol with others. Participants associated this type of drinking with “not knowing how to solve problems,” being “embarrass [ed] to ask help,” or “not [having] any trustful friend” (Carmen Alto, male participants).

A dominant theme identified through our analysis was captured by the recurrent trope *“No hay control”* (There is no control), which was used to represent the multiple ways in which the reported shifts in patterns and motivations for problem drinking were attributed to various forms of control or absence of control, including control of parents over their children, men’s control over the household and self-control by drinkers, the absence of control by the government over the growing local alcohol market.

### Parents’ lack of control over their children’s alcohol use

When asked about the general alcohol use in the community, most participants raised concerns over adolescent alcohol use. Participants repeatedly stated that “children are abandoned,” attributing underage drinking to a lack of parental control and supervision. Others said that children drink with friends to create a substitute family situation to counteract feelings of loneliness and absence of parental emotional support.

Many participants from Carmen Alto explained that this precarious family environment involved children relocating to urban areas for the purpose of education while their parents remain in rural areas. Relocation of children for education in Socos is not as common as in Carmen Alto. In Socos, parents often embark on short-term travel to the *selva* (the Amazonian jungle) or to other cities to earn money to meet household economic needs. The children then live with relatives such as cousins and grandparents. During fieldwork in 2016, a 15-year-old girl died after being sexually and physically assaulted by several intoxicated adolescents at a party in a neighbouring district. The assault occurred in a house with no adult supervision or presence. This incident was often mentioned as an example of how children are placed in insecure situations without parental care, supervision, or control. Young community participants, health workers, and police officers in Carmen Alto often referred to *fiesta semaforo* as a facet of contemporary youth culture. Specifically, male and female adolescents invite each other to underground parties involving excessive drinking of cheap liquor mixed with water or soda (i.e., *combinado*) and sexual activity using secret codes transmitted by cellphone and Facebook to avoid the attention of parents and authorities. During one focus group in Carmen Alto, participants described this context:

P6: It has been an invasion [of land], parents have come [as] they wanted to occupy a plot of land, and they have brought their children. They brought their kids here so that the kids can study. And then parents are in *chacra* (field). Who is going to control this kid?

P7: No one.

P5: When a friend comes, and then they go to drink.

P4: They go to fiesta, and then they drink.

P5: Disco.

P6: This largely happens in this community.

(Carmen Alto, female, focus group)

Persistent economic hardship forces parents to work long hours, which results in separating parents from children and/or limiting their time together. One 48-year-old female focus group participant from Carmen Alto stated: “I have seen mother and father are dedicated to *chacra* (plot of land). They are gone at 4 o’clock in the morning because of the lack of an economy”.

Participants further stated that, compared with their own upbringing, parents are having a hard time controlling their children. Participants noted that in the past parents and elders used *chicote* (whip) to inculcate obedience and respect, and this was considered useful to “give good orientation” (Socos, male, age 71, interviewee). Participants reported that children were today becoming “rebellious and dominate their parents” (Carmen Alto, obstetrician, interviewee) by referring to human rights and knowledge of institutional protection. It was noted that this situation contributed to youth alcohol consumption. “Parents were previously stricter and never let children go out in the night” (Carmen Alto, male, age 20, focus group). Most participants still largely considered the *chicote* as an effective and acceptable disciplinary tool. In this context, “DEMUNA” and “law” were frequently cited as barriers for parents to control their children’s alcohol use. DEMUNA (the Municipal Office of the Ombudsperson for Children and Adolescents *(Defensoría Municipal del Niño, Niña y Adolescente*) was established in 1993 to promote and protect the rights of children and adolescents. It has the responsibility to intervene any time the human rights of children and adolescents are violated. In December 2015, Peru also adopted a law prohibiting the use of physical and other humiliating punishment with children and adolescents (No 30403 *“Ley que prohibe el uso del castigo físico y humillante contra los niños, niñas y adolescents”*). The law, social services, and intervention by DEMUNA to protect women and children from violence were largely appreciated. A 60-year-old female focus group participant in Socos stated: “Now the law prevents men from hitting women”. At the same time, others see some parents having difficulty controlling their children’s misbehavior because the parents fear that their children will report them to DEMUNA, claiming that their parents’ discipline is an abuse of children’s rights.

“I think now we cannot pressure children. They drink and say ‘[If] you say something, then I am going to report you [to DEMUNA]’ and mother has this fear of being accused.”

(Socos, nurse, interviewee)

### Drinkers’ lack of control over themselves

#### Men’s problem drinking explained by machismo

Lack of control was also mentioned to describe the way adult male drinkers, who were initially happily drinking and dancing, start to express their problems, cry, or become aggressive, sometimes to the point of violence, and end up falling asleep on the street until the next morning. It was not uncommon to see security guards or other community members intervening when male intoxicated drinkers started to argue and fight during the community fiestas and *recreos*.

Many participants referred to the cultural concept of *machismo* to explain this “little control with respect to male drinkers” (Carmen Alto, psychologist, interviewee). *Machismo* was generally cited to describe gender inequality in current everyday life. In reference to drinking specifically, community participants, police officers, schoolteachers, and health professionals noted that *machismo* was related to how men justify their drinking practices, spending ‘hard-earned money’ on drinking as an entitlement from being a ‘breadwinner’. The common answer to the question, “Who drinks more? Women or men?” was “The one who has more money; men.” (Socos, male, age 20, focus group). The Andean way of sharing drinks is entangled with the practice of *machismo*. During a bout of drinking, men show not only physical toughness by the amount of consumption but also by their ability to purchase drinks for others.

When competitiveness accelerates drinking, controlling drinking and spending becomes challenging. A young male focus group participant in Socos stated: “Before you become drunk, you think. When you drink, the alcohol [level] goes up, and you start losing control of yourself. You practically forget everything for the moment.... Now the experience is that while you are drunk, you could be what you wish to be without control....”. Many male community participants stated that they often feel regret (*arrependimiento, pesares*) and guilt (*culpa*) the following day when they remember what they did and how much they spent on alcohol the previous night.

Facilitator: What do you do after drinking? What do you do on the following day? How do you feel and what do you do?

P3: Take a cold shower.

P12: Reflect.

Facilitator: What do you reflect on? About why you drank?

P12: Why … .yeah, about what kind of things I have done. I remember, maybe I may have done something bad or I was not respectful to someone, or maybe, I misbehaved, how many cases I drank.

P1: How much I spent.

P12: How much I spent.

Interviewer: Yes, like damaging the economy of your family?

P12: Yeah, I analyze whether I have damaged, maybe … [whether] I drank for free while friends spent all the money.

(Socos, male, focus group)

A common practice among Andean individuals engaged in labor-intensive agricultural work is to regularly drink a “minimal proportion” of alcohol to “feel motivated to continue working”. In contrast, drinking a “maximum proportion [of alcohol] can result in economic loss” (Carmen Alto, female, age 31, focus group). One 48-year-old male focus group participant in Carmen Alto stated: “If you do not control alcohol, alcohol controls you. For this reason, it is necessary to know up to what point you can drink. There are others who do not have this kind of control.” On one hand, the duration of a bout of drinking and the total number of drinks consumed “depends on the money” (Carmen Alto, male, age 19, focus group) and “If you have money, you can [continue drinking] till dawn or continue” (Carmen Alto, male, age 20, focus group). At the same time, men try to exhibit control over the household economy and their health by “calculat [ing] the quantity of beer” they pour into the glass, as they know that it is their turn to buy another round of drinks if they empty the bottle (Carmen Alto, male, age 50, focus group). They know “who buys and who does not buy”, while being seen as a free rider (i.e., only drinking alcohol purchased by others) has a risk of “not being invited for the next time” (Carmen Alto, male, age 50, focus group).

#### Norms of consumption and household control

The gendered division of labor in the family seemed to excuse men’s lack of control over their alcohol consumption. Young male focus group participants from Carmen Alto considered that married women with children drink less than single women because the former are responsible for taking care of children. During the fieldwork, the first author frequently witnessed women drinking with men during community fiestas and family gatherings. Most women consumed only a small amount of alcohol and often left earlier than men, suggesting that their alcohol use, while frequent, is distinct from that of the men. Female intoxication is largely frowned upon, as a nurse in Socos stated: “We see men being *borracho* (intoxicated) but seeing some women *borracha* is bad.” With this cultural norm, a 60-year-old female focus group participant in Socos mentioned, “some couples drink together and the wife needs to control herself”.

The apparent lack of control in men’s drinking reflects not only excessive alcohol use but also men’s identity-making and power representation in public and domestic spaces. In public spaces, the type of alcohol drinks can mark the socio-economic status. In both districts, *chicha* (fermented corn beer), commercial beer, *caña* and *trago* (alcohol mixed with water) are commonly consumed. *Chicha* is largely consumed as a ritualistic part of community fiestas, while drinkers of cheap *caña* and *trago* are commonly perceived as those with limited economic resources, who are often *campesinos* (rural peasants). In addition, some men are reported to spend a disproportionate amount of money on alcohol, often to the point of spending all received wages during a weekend or life savings at a *fiesta patronal*, even though both they and their spouses work to cover household expenses. While participants often joked that “people work to drink” in Ayacucho (Socos, male, age 21, focus group), spending earnings on alcohol rather than on household expenses as a demonstration of manliness was questioned and considered a lack of responsibility. Particularly evangelists, health professionals, and high school teachers characterized men’s spending on alcohol consumption to be reckless extravagance in contradiction with the economic situation of Ayacucho where some “children are still without bread” (Socos, male, age 63, focus group).

In the domestic space, men’s spending money on alcohol was criticized for the negative impact it had on the household economy, potentially leading to conflict between spouses. Some female participants showed their frustration with their husbands’ spending on alcohol by describing that “It is worse when a drunken husband responds, ‘I do not drink with your money’” (Socos, female, age 48, focus group).

Health workers and high school teachers often cited the concept of “*cultura etílica*”, which expresses an individual’s responsible drinking and self-control. A psychologist explained that *cultura etílica* is “control, [with awareness that] I am drunk and I can cause some problem because I know how I am,” unlike “others [who] have many responsibilities the next day and know how they are but they do not control” (Carmen Alto, psychologist, focus group). Among community participants, it is also commonly understood that people “should drink rationally (*racionalmente*) only up to the point [they] can afford” (Carmen Alto, male, age 48, focus group).

P5: Instead of spending money on alcohol, it is better to eat.

P4: It is better to eat no matter how little.

P6: You should fill yourself up with 100 Soles, not with beer.

P4: Sometimes we spend in vain.

Julián: Spending in vein

P3: When we are drinking with friends, we challenge ourselves. And then we use our money and nothing remains for our wife.

(Socos, male, focus group)

### Lack of control over alcohol supply

#### Commercialization of consumption

Participants also noted the normalization of drinking described as *costumbre* (custom, habit), which was attributed to the lack of control over the expanding alcohol market. One young male focus group participant in Socos described this expansion by stating: “If you leave [home], you see people drinking on the street, [and] at the corner. There is no respect … At one corner, you see one fiesta, and at another corner, you see another.” The easy availability of alcohol has also shaped drinking practices in the community. While the traditional community fiestas still play a role in strengthening social relations through the practice of Andean reciprocal support, *ayni*, many participants no longer attributed to fiestas the values and meanings that they once held. For instance, a schoolteacher in Carmen Alto stated:

[Traditional] fiesta is commercial. Now it is already a commercial activity because now everything is about selling. For example, I see people want to make money, profit in the *fiesta patronal* … Yes, it used to be something cultural, but in the past few years, it is not any longer. The economic aspect is added. Profit.

(Carmen Alto, schoolteacher, focus group)

Many community participants shared this sentiment. One elderly male participant in Socos described the organizing of traditional fiestas of Catholic Saints as being costlier as it becomes “more modern”, stating that nowadays, “Only the one who has money can be *mayordomo* (host of the community festival),” who “takes the responsibility [of hosting the fiesta] with money with caprice [not with faith]” (Socos, male, age 71, interviewee).

On one hand, in resource-scarce rural settings in the Andean highlands like Socos, community fiestas with collective drinking are still considered to be a practical mechanism to sustain subsistence living by strengthening and maintaining the bond of relatedness among family and community members. One 50-year-old female focus group participant in Socos stated: “In *techada de casa* (house building), we help each other and drink and dance, this is how we build *casa* (house). In *techada de casa*, we become close to each other, drink, if we do not do it, there is nothing.”

Participants in both districts often compared the availability of alcohol to staple food. As one stated: “Bread can run out, but beer is always [available] at each store, it is ever-present” (Carmen Alto, female, age 43, focus group). In addition, participants in both districts noted the increase in drinking venues such as *recreo* and events such as festivals and music concerts, the improved road conditions and accessibility of transportation between Ayacucho and Socos, and the availability of low-cost alcoholic beverages mixed often with soda drinks.

Participants described the expansion of the local alcohol market by saying: “Beer is a gold mine”, and “Everyone now wants to open his *cantina* (bar) all over the world” (Carmen Alto, male, age 20, focus group). Despite the poor economic conditions, alcohol is affordable and accessible. This includes *caña* (sugarcane alcohol) and *trago* (alcohol), which are thought to make people drunk rapidly and be harmful to health. One nurse in Socos stated: “*caña* damages our brain more and [it is better] to choose beer.” These types of alcohol can be easily found at local vendors who also sell alcohol on credit to regular customers, facilitating access even to those who have limited resources. The diversification of alcoholic beverages was also noticeable; as one 43-year-old female focus group participant from Carmen Alto stated: “Now those businesses are innovating that theme of beer. Now there is beer made of *quinua* (quinoa) and *trigo* (grain)”. Also, when the first author returned to Ayacucho in March 2019, she saw Budweiser, which had become available in the Peruvian market in 2017 [45], being sold for the first time during Carnival.

Participants often stressed how advertising by alcoholic beverage companies and media created demand for alcohol. There are no laws restricting alcohol advertising or promotion in Peru [1]. Participants often explained the perceived increase in alcohol use among adolescents and women from the influence of advertising through a wide range of media.

P10: And the advertisement also influences a lot. For example, on TV, we see young girls drinking, right? In the TV commercial, we see women drinking...

P3: In the advertisement, we see girls drunk and you think that they are cute.

P10: There are women and young girls who are becoming like that. Those women also buy beer to drink with men.

(Socos, health professional, focus group)

An advertisement for Pilsen beer that proclaims “Thursday is a day of buddies” [*Jueves de Patas*] is another frequently cited example. Participants were conscious that this was a marketing strategy to add an occasion to drink during the week, as drinking on the weekend with colleagues, friends, and family members is already an established practice.

P5: Cheap alcohol drinks that do not even have any brand or any registration, I think, need to come from the top [government] to avoid [unofficial] factories of those alcohol. We need to cut these alcoholic beverages. Up to now they do advertisement for *“Jueves de Patas”* (Thursday for buddies) and *“Viernes de Amigos”* (Friday for friends).

P7: Fathers’ day, Friends’ day, everything [about drinking is] everywhere in the city.

P6: From the same manufacturer. *Jueves patita* ha ha ha …

P5: Because everything about this is to motivate drinking.

(Socos, high school teachers, focus group)

With the felt impact on alcohol availability, the alcohol industry is blamed for creating alcohol-related problems in the community while making profits off of consumption. Participants repeatedly mentioned Peru’s largest brewery, Backus, as making huge profits: “[Alcohol] business is making millionaires, Backus is a millionaire” (Carmen Alto, male, age 20, focus group).

#### Capitalizing on vulnerability

Parallel to excessive alcohol use at community fiestas and weekend social gatherings, participants expressed that alcohol use can be a means of coping with psychological distress for “some [who] do not know how to manage problem and seek alcohol” and “think that they can solve problems while being drunk” (Carmen Alto, male, age 20, focus group). They described solitary drinkers—those who drink as a means of coping with hardships such as family conflict, financial concern, and breakup who gradually develop *alcoholismo* (alcoholism). Some participants suggested that the alcohol companies take advantage of this vulnerability to pursue profits. Community participants framed problem drinking as the result of market forces that push the rural poor unable to find a solution to their problems to consume alcohol.

Previously, possibly in the 80s and 70s, here in our community, people used to celebrate, organize activities with the purpose of reevaluating our *costumbres* (customs, habits). Day by day, they do not organize fiestas with this purpose. We do fiestas with the purpose of getting people to consume alcohol, and this is the purpose of business. Those who benefit are only big business. How much revenue do we generate for Backus? Whatever quantity, people like, people like to organize fiestas, every weekend, fiesta in Arenales, [fiesta] in San Luis, now for the Day of All Saints. We have a number of fiestas. Then, those businesses benefit, and we do harm to ourselves, people in the rural area. People who are not prepared. People who cannot solve their problems dedicate themselves to alcohol. “I drink to take refuge in alcohol” [they say].” (Socos, male, focus group)

The active promotion of alcohol for economic gain was contrasted with the economic hardships and precarity of those who were consuming alcohol. This sentiment was summarized by one participant, who stated: “Some people who have economic problems take refuge in alcohol even though they do not have [money] to buy other types of things” (Socos, health professional, focus group).

#### Absence of government control

Alcohol sales have been increasing in Peru, exemplified by a 24.6% increase in Backus’s sales of beer from 2012 (S./3160.2 mil) to 2017 (S./3939.0 mil) [45, 46]. Community participants often blamed the local government for not tightly controlling the sale of alcohol to minors or the sale of illicit alcohol beverages, and for not limiting the hours of the day when alcohol is sold. Alcohol vendors who were interviewed informed us that they complied with the law, however. While witnessing *alcohólicos,* those who use alcohol as a means of coping with suffering, some community participants continue to blame the government. A female focus group participant in Carmen Alto stated:

An *alcohólico* doesn’t seem to become alcoholic because he wanted to, but there are reasons. They can be infidelity, others can be a factor related to work, lack of economic resources, and if he has a family, the more he drinks, the more fights [due to] the consumption of alcohol … Yes, the authorities are to be blamed for allowing stores to sell alcohol … there is ethyl alcohol which they sell, mixed with water, and that is what causes them to become ill, and that really gives me *pena* (sadness), when I see an *alcohólico …*

Participants associated this lack of government control with the benefits they received from an expanding alcohol market.

With this drinking including [drinking by] minors, when people drink, there is more revenue for the government because of more consumption. Although people say there would be pills, medication [for addiction], the government will not approve it because there is more revenue for the government wherever there is a factory for alcohol, or even coca. For this reason, drinking will not disappear.

(Socos, male, age 54, focus group)

The local government collects tax from flourishing business activities in the entertainment zone of Carmen Alto. One Saturday night in Carmen Alto, I saw many women opening street stalls in front of bars and *recreo* to sell food to hungry drinkers, while paying the municipality some monthly fee to use the street for their business.

## Discussion

The local perspectives described above highlight the complex patterns of power and meaning exerted and experienced by different actors at different levels, which shape behaviour and local understanding regarding loss of control over alcohol. Many of these sites of control, which have been maintained by static power hierarchies at family and community levels, have shifted dramatically with the globalization of markets and cultures, and the influence of transnational alcohol companies. One high school teacher in Socos described a growing consumer culture and circulation of ideas and images linked to transnational markets in students’ clothes and music [47, 48]. When drinking occasions were limited to traditional fiestas based on Andean agricultural cycles and the religious calendar, they were valuable opportunities for sustaining the safety net of reciprocity in goods and services within a community. Today, motives of alcohol use are not necessarily tied to historically meaningful events, celebrations, or functions, resulting normalizing collective drinking including adolescent alcohol use.

In this context, some health professionals stressed the lack of self-control as a driving factor of current alcohol use in Ayacucho. This aligns with a common perspective that attributes excessive alcohol use to a failure in self-management. However, there is also evidence that this perspective risks subjecting the drinker to stigma and social marginalization [16, 19]. Importantly, our study highlights that structural and cultural dynamics contribute to consumption practices. Singer’s political economy approach positions alcohol use in relation to social suffering created by macrolevel structures and relations of power such as socio-economic inequalities, systemic discrimination, and dominant corporate institutions [38, 49]. Furthermore, historical understanding of social and economic structures sheds light on the way conditions of structural violence normalize affliction [50, 51].

Reference to local history demonstrates a change in drinking practices in the Andean highlands of Peru tied to structural vulnerability. In Ayacucho where persistent economic hardship is endemic, past and present struggles intersect with future uncertainty to create conditions conducive to alcohol use as a coping strategy [40, 47–49]. A change in drinking practices started to be seen in the l970s where greater access to cash and credit turned occasional drinking into a routine behaviour at the same time that alcohol was being promoted more actively and was more accessible [52]. As the government failed to simulate economic development in the Andes through the Agrarian Reform, some explain increased consumption among economically vulnerable rural *campesinos* (peasants) as a means of coping with frustration and disillusionment due to limited economic opportunities [24].

Furthermore, the period of the extensive political violence from 1980 to 2000 that resulted in grave psycho-social consequences appeared to shift the meaning of ritualized drinking into a mode of coping and healing. In Carmen Alto, many villagers fled the intensified political violence occurring in rural areas of Ayacucho. Some participants reported that alcohol had been consumed to take refuge *(refugiar)* by those who had difficulty adapting to a new urban environment that represented a different way of living from rural subsistence farming. A schoolteacher also reported the increasing alcohol use among youth gangs (*pandillas)* who felt abandoned due to the loss of their parents during the political violence and family disintegration after that period. In rural areas like Socos, some people were seen to start drinking to forget sadness and reduce constant fear from death, while sleeping in caves and moving from one place to another to hide from the threat of violence. In the post-conflict environment where staying silent and forgetting the painful past became a way to live peacefully, alcohol use was an Andean cultural practice that functioned as a strategy to achieve this objective [26, 53, 54].

Today, while the popularity of commercial beer is growing, artisanal beverages such as *chicha*, *caña* and *trago* are still commonly consumed. *Caña* and *trago*, which are available at a lower price than beer, are often consumed by drinkers who are developing *vicio* and/or and people with low economic resources. An analysis of the content of several samples of alcohol beverages sold in Ayacucho revealed that these cheap alcohol beverages are often contaminated with fusel alcohol, a by-product of alcoholic fermentation (Pedersen D: Estudio sobre patrones de consumo de alcohol en poblaciones urbanas y rurales de la sierra (Ayacucho, Peru), unpublished). Alcohol beverages often illegally consumed by youth is also based on low quality ethanol mixed with soft drinks or powdered juices and harmful to health [48, 55].

Alcohol use by “abandoned” children reflects the long-standing socio-economic inequality between the highlands (33.3% poverty rate) and the coast (14.3%) that many of the Andean families have faced [56, 57]. These disparities have been closely linked to racial hierarchies where indigenous populations are placed at the bottom [58]. Government policies since the 1960s have largely neglected rural highland communities in Peru [58]. Today, in Ayacucho, particularly in rural areas like Socos, many families are supported by the government cash transfer program (*Programa Juntos*). Due to persistent poverty, some parents and children must be separated, as parents are using the available resources not only to meet immediate family needs but also to invest in children’s education in order to help the children access other economic opportunities [59]. The outcome of this separation is the limited shared family time and space to cultivate collective well-being, *“sumaq kawsay,”* shaped through cohabitation by accumulating mutual trust, help, and respect among family and community members [60].

In addition, precarious family relations may detract from parents’ control over children’s alcohol use. Because of parents’ migration and/or children’s relocation, building family trust and bonds can become difficult. In this situation, the knowledge that children acquire through education about their rights and the institutional instruments to protect these rights seems to have an unintended effect on the traditional mode of discipline, in which parents would control children’s misconduct. The child protections from the state are enmeshed with these shifts in family composition, connectedness and the added pressures of economic precarity.

Participants also emphasized how alcohol consumption was tied to forms of control exerted by men in households using the cultural concept of *machismo*. They construct their masculine identity among their peers, family, and community by practicing a right to spend lots of money on alcohol that is reserved for breadwinners. While the definition varies, *machismo* is generally associated with competition against other males and the expression of ‘masculine’ norms [61, 62]. Being macho is typically presented through an ability to drink alcoholic beverages in large quantities without giving in to the effect of intoxication [61]. Likewise, men’s heavy episodic drinking is a common drinking pattern, as 90.5% of male drinkers in Ayacucho are found to consume five or more drinks at one or more occasions, compared with 74.0% in Lima [63]. Work allows men to demonstrate their manhood symbolically by not only providing for the basic needs of their families, but also by participating in the gatherings. Casual laborers and construction workers often go out drinking “out of happiness when they receive wages” (Socos, female, focus group) and in some cases, artisanal illicit alcohol products such as *caña* are included as part of the work package [55]. In these collegial spaces, peer pressure usually drives men to spend more money than initially planned on alcohol to validate their own masculinity, while achieving cultural values of connectedness [62].

On the other hand, during intoxication, men’s demonstrated power is juxtaposed to their lack of control over their feelings or their life circumstances. Several female community participants made fun of their partners’ crying when drunk, describing it as an expression of the rarely disclosed sadness, anger, and frustration that comes from their inability to solve their problems. SY witnessed several men who at first cheerfully danced during the *fiesta patronal* in Socos, but then started to weep after drinking for several hours. Drinkers’ crying and language of despair reflects the experience of poverty and suffering [64, 65]. Not being able to fulfill the traditional role of breadwinner or to exercise masculinity due to poverty is a source of frustration among Andean men in Peru, and places pressure on families [66]. While men’s public crying contradicts existing gender norms, this contradiction may be understood as a “time out” conceptualized by MacAndrew and Edgerton. They argue that getting intoxicated allows the drinker to behave in ways that are not normally socially acceptable [67].

The conditions of violence, economic precarity, and weak government control over alcohol markets have facilitated alcohol consumption. The alcohol industry has directly and indirectly taken advantage of weakened state capacity and control in this unstable political environment [68]. While the personal and cultural conditions contributed to the current state of alcohol consumption, the “aggressive” sale of *alcohol metílico* (methyl alcohol) is believed to explain the dramatic shift in the meaning and practices of alcohol consumption in post-conflict Ayacucho [26]. Participants’ recurrent references to Backus, which was founded by two Americans in Lima in 1879 and has grown to be the largest brewery with seven production sites in Peru after merging with three other breweries in 1996, indicates the extent of its visibility and influence [69]. SAB-Miller—one of the top international brewing companies to which Backus once belonged—ranks among the top 10 largest advertisers in Peru [4]. After becoming part of the international AB Inbev group after its merger with SABMiller group in 2016, Backus has diversified by selling global brands such as Budweiser, Corona, and Stella Artois [70]. In 2018, this strategy is reported to have increased Backus’ gross sales by 73% and profits by 63% from the previous year [71].

Community participants are experiencing how Backus’ monopoly of the alcohol industry profits from existing norms of collective drinking. Their perceptions and practices reflect one of the three marketing pillars of Backus, namely to create a sense of “belonging” by tapping into Peruvian history, cultural heritage and traditions to promote beer culture among the youth [72]. Meanwhile, the local government does not appear prepared to actively control the sale of alcohol due to the revenue from alcohol consumption and related businesses. At the national level, although none of the participants provided concrete evidence, the appointment of Fernando Zavala, the CEO of Backus since 2013 and also the deputy economy minister (2002–2005), as Prime Minister in 2016 is perceived to reflect the close connections between the Peruvian government and its largest brewery [73]. The alcohol industry could exert undue influence on decision makers at the highest level of government public health policies and regulations to dilute its commitment to making problem drinking a public health priority [2]. In fact, Backus has been financing the public infrastructure projects in different parts of Peru under the Law 29,230 (*Lay de Obras por Impuestos*). This law aims to promote private investment in infrastructure in underdeveloped areas through public-private partnerships, where private companies can select interested projects/areas, develop relations with groups at the municipal/regional level, and improve the corporate image [74].

At a macro level, the dominant neoliberal paradigm has shaped how government, either directly or indirectly, influences the supply of alcohol in a global market system [18]. While people often overlook “commercial determinants” of alcohol consumption, the alcohol industry’s aggressive marketing in many LMICs can exploit the poor legislation and regulations and shape people’s drinking patterns and behaviours [2, 7, 8]. These “corporate social responsibility” initiatives provide power to frame problems, gain legitimacy, and shape public policy [75, 76]. In Peru, the illicit market (including artisanal drinks) is said to account for nearly 30% of all consumed alcohol beverages [55]. Backus has been actively promoting the reduction of illicit alcohol products through public campaigns [55, 71, 77]. By blaming the illicit alcohol market, Backus has attempted to promote a positive corporate image while creating room to promote their own products. Backus has also bolstered its public image through ‘harm reduction’ campaigns. For example, the company launched a campaign of “responsible drinking” in 2018 during *Semana Santa* (Holy Week), a traditional religious event for drinking during consecutive days [78]. Overwhelming evidence suggests that such responsible drinking messages are strategically ambiguous and are implemented to increase sales and strengthen public relations [79]. Such campaigns also shift the responsibility for consequent harm to the consumer [76].

While the market, social, and governance forces can contribute to alcohol-related illnesses that also stigmatize and isolate drinkers, the notion that “*alcoholismo* is a social illness,” as stated by a schoolteacher, highlights the importance of incorporating the voices of community members into the local action for preventing harmful alcohol use. In the Andean socio-cultural context, where collective drinking has its own cultural meanings and social values, change needs to be driven by people in the community. One psychologist in Carmen Alto stated: “Change or interven [ing] [is difficult] because this is very much a theme of culture or socio-cultural. It is ingrained … To say people ‘Do not do’ is like [saying] ‘Stop being Peruvian. Be Chilean.’” This relationship between culture, identity and the political and economic conditions is an important aspect of intervention. This analysis has illustrated that the conditions of consumption intersect at different levels to condition environments of consumption, while the moralistic narrative of responsible drinking and control can distract from the social, economic, and policy conditions that lead to consumption [80, 81]. People with limited socio-economic resources such as subsistence farmers, daily laborers, and youth, are positioned in an inferior socio-economic status within prevailing hierarchical relationships of power, leading to physical and emotional suffering [41, 50]. Easily accessible alcohol often becomes a means of coping with their daily struggle and psychological distress. Furthermore, the absence of government control creates conditions for alcohol companies to suffuse and shape cultural practices towards market aims while legacies of violence and economic precarity further condition consumption practices.

### Strengths and limitations

The political economy approach adopted in the study unpacked complex macro-micro interactions and power dynamics that constantly shape the meaning and practice of alcohol use in the south-central Andean highlands of Peru. A major limitation of the current study is one of scope, namely the limited exploration of the role of religion in the popular perspective on control. In the Andean highlands, evangelical churches associate alcohol use with backwardness, laziness, and lack of self-control, while playing a role of healing site of a range of illnesses including *alcoholismo* [65, 82]. In both study sites, evangelism that prohibits the consumption of alcohol has had a growing influence on community activities since the political violence took place [83]. In focus group discussions, evangelistic community members shared their experiences—such as having a drinking problem before conversion from Catholicism, or having no experience of drinking alcohol. Even though some conversions back to Catholicism were not uncommon, evangelists often stressed how abstinence can bring health benefit, family harmony, and community safety. Likewise, evangelists are sometimes seen as “criticizing those [Catholic residents] who drink alcohol and cause problems” (Socos, male, age 60, focus group). With this potential tension between Catholic and evangelist participants, the presence of Catholic community members in the focus group may have inhibited evangelistic participants from generating a distinct faith-based perspective on control.

Furthermore, female participants might have had difficulty sharing their perspectives on and experiences of alcohol use with other participants and/or research team members. While male and female participants were separated during focus group discussion, the existing cultural norm that female alcohol use outside of community fiestas and family gatherings is not widely acceptable, particularly in Socos, may have constrained the access to the full range of female participants’ accounts. The cultural significance of female alcohol use and its impact on power relations and family dynamics in the present research setting and other LMICs could be deepened in future studies.

## Conclusion

In this paper, participants’ perspectives illustrate how problem drinking is shaped not simply by an individual drinker’s lack of self-control but also shaped by an environment that enables the unrestrained marketing of alcohol products and the creation of a culture of consumption. Harmful consumption is mediated by the reshaping of the Andean cultural practice of collective drinking. Attending to local perspectives is essential for policies and interventions that connect structural dynamics with the cultural and experiential aspects of alcohol consumption.

## Data Availability

The data from interviews are available from the corresponding author on reasonable request.
